# Effect of propolis extract and chlorhexidine in treating chronic periodontitis: A randomized controlled trial

**DOI:** 10.6026/973206300214179

**Published:** 2025-11-15

**Authors:** Darshana Digambar Shivatare, Vidya Dodwad, Nishita Bhosale, Devashri Newaskar, Manasi Yewale, Roopal Gupta

**Affiliations:** 1 Department of Periodontology, Bharati Vidyapeeth (Deemed to be University) Dental College and Hospital, Pune, Maharashtra, India

**Keywords:** Chronic periodontitis, scaling and root planing, chlorhexidine, propolis, subgingival irrigation, local drug delivery

## Abstract

Chronic periodontitis remains a significant dental health issue, with current treatments often relying on chemical irrigants like chlorhexidine, which may have limitations. Therefore, it is of interest to compare the clinical and microbiological effects of 0.2% chlorhexidine (CHX) and 25% propolis extract as subgingival irrigants adjunctive to scaling and root planing in chronic periodontitis patients. Twenty patients were randomly assigned to receive either CHX or propolis following SRP. Significant improvements were observed in clinical parameters (Gingival Index, Plaque Index and Probing Pocket Depth) and microbiological reduction in both groups, with propolis showing superior results. Propolis extract exhibited greater efficacy in reducing clinical symptoms and bacterial detection, suggesting it as a promising natural alternative to CHX.

## Background:

An assortment of periodontal infections includes chronic inflammatory conditions affecting the supporting structures of teeth, gingiva, periodontal ligament, cementum and alveolar bone [1]. Among these, chronic periodontitis is the most prevalent form, marked by alveolar bone resorption, attachment loss, periodontal pocket formation and gingival inflammation. It represents a major cause of tooth loss in adults worldwide and has significant implications on oral health, mastication, esthetics and overall quality of life [2]. Global estimates reveal a burden of 951 million cases of periodontal disease in 2021, underscoring its pervasiveness. In India, pooled data indicate that around 51% of adults are affected by periodontal disease [3]. The commencement and continuation of periodontitis is primarily associated with the accumulation of dental plaque biofilm containing diverse bacterial species. These microbial communities interact with the host immune system, leading to an exaggerated inflammatory response [4]. Host-derived mediators, including cytokines, prostaglandins and matrix metalloproteinases, result in connective tissue destruction and alveolar bone resorption [5]. Key pathogens such as "*Porphyromonas gingivalis*, *Aggregatibacter actinomycetemcomitans* and Prevotella intermedia" have been strongly implicated in disease pathogenesis [4]. "Scaling and root planing (SRP)" persists to be the benchmark for managing chronic periodontitis, aiming to mechanically remove microbial deposits, calculus and endotoxins [6]. While effective in reducing probing depths and improving clinical attachment, SRP has limitations: incomplete removal in deep/anatomic sites, persistence of pathogens within tissues and rapid recolonization. This has motivated the development of adjunctive antimicrobial approaches [7]. Systemic antibiotics like tetracyclines and metronidazole can enhance SRP outcomes but raise concerns of antimicrobial resistance, gastrointestinal effects and compliance [8]. Consequently, local drug delivery (LDD) systems-such as gels, chips, fibers and irrigants-are favored for targeted antimicrobial action with reduced systemic exposure [9]. Among LDD agents, chlorhexidine (CHX) is extensively utilized due to: Broad-spectrum antibacterial efficaciousness against Gram-positive and Gram-negative bacteria, fungi and viruses [10]. In dentin, this residual antimicrobial activity has been demonstrated for up to 21 days [11]. Propolis, a resinous substance produced by honeybees, is rich in flavonoids, phenolic acids and aromatic compounds, offering antimicrobial, anti-inflammatory, antioxidant and immunomodulatory benefits [12]. Studies show its inhibitory effects on periodontal pathogens like *P. gingivalis* and improvement in periodontal outcomes, such as reduced probing depths. Its safety and biocompatibility make it an appealing natural adjunct in periodontal therapy [13]. Despite promising findings, direct comparative studies evaluating propolis versus chlorhexidine as subgingival irrigants in chronic periodontitis are limited. Consider the demand for safer, natural and cost-effective adjuncts in periodontal therapy. Therefore, it is of interest to determine the fill that gap by comparatively assessing 25% propolis extract and 0.2% chlorhexidine solution as adjuncts to SRP, focusing on both clinical and microbiological outcomes.

## Materials and Methodology:

The present study was designed as a prospective, single-blind, randomized controlled clinical trial conducted in the Department of Periodontology, Bharati Vidyapeeth (Deemed to be University) Dental College and Hospital, Pune, Maharashtra, India, with a study duration of one month. The trial aimed to compare the clinical and microbiological efficacy of 0.2% chlorhexidine solution and 25% propolis extract as subgingival irrigants adjunctive to scaling and root planing (SRP) in patients with chronic periodontitis. The research protocol was examined and authorized by the Institutional Ethics Committee. Each participant received comprehensive information about the purpose, methodology, potential risks and benefits of the study. Written informed consent was obtained from each subject prior to participation. "Subjects were recruited from patients reporting to the Outpatient Department of Periodontology, Tertiary care hospital, Pune." Inclusion Criteria included was age group: 25-60 years. "Systemically healthy individuals", "Patients" diagnosed with "mild to moderate generalized chronic periodontitis" according to the "American Academy of Periodontology (1999)" classification. The inclusion criteria for the study were as follows: participants aged between 25 and 60 years, who were systemically healthy and diagnosed with mild to moderate generalized chronic periodontitis according to the American Academy of Periodontology (1999) classification. Additionally, participants had to have periodontal pockets with a probing depth of 4-6 mm in at least one tooth and must be willing to participate in the study and provide written informed consent. Exclusion Criteria included patients with systemic diseases or immunocompromised conditions. The exclusion criteria for the study were as follows: patients with systemic diseases or immunocompromised conditions, a history of allergy to chlorhexidine or bee products, or those who had undergone surgical or regenerative periodontal therapy within the last 6 months. Additionally, participants who had used systemic antibiotics or antimicrobial therapy in the past 6 months, pregnant or lactating women, and patients with deleterious habits such as smoking, smokeless tobacco chewing, or betel quid use were excluded from the study. A total of 20 participants were enrolled in the study, with 10 participants assigned to each group. The sample size was calculated to provide a statistical power (β) of 80% and a significance level (α) of 0.05 for detecting inter-group differences in clinical and microbiological parameters. Randomization was done on a 1:1 ratio using a simple coin flip method. Subjects were randomly assigned into two groups: "Group A (Control Group): 10 participants received subgingival irrigation with 0.2% Chlorhexidine solution. Group B (Test Group): 10 participants received subgingival irrigation with 25% Propolis extract (Super Bee Propolis tincture, Hi-Tech Natural Products India Ltd., India)". To minimize bias: One calibrated examiner performed all clinical recordings. Another operator performed subgingival irrigation. Participants were blinded to group allocation. At baseline, day 15 and day 30, the following parameters were assessed: Gingival Index (Löe and Silness, 1963): Measures gingival inflammation severity (scores 0-3). "Plaque Index (Turesky-Gilmore-Glickman modification of the Quigley-Hein Index, 1970)": Assesses the thickness of plaque on tooth surfaces. Pocket Probing Depth (PPD): "Measured using a UNC-15 periodontal probe, at six sites per tooth (mesiobuccal, buccal, distobuccal, mesiolingual, lingual, distolingual)". All measurements were recorded by the same examiner to ensure reliability. Subgingival plaque samples were obtained from the deepest periodontal pocket (4-6 mm) in each selected tooth. After isolating the area and removing supragingival plaque, a sterile Gracey curette was inserted into the pocket to collect plaque. Samples were immediately transferred into 2 mL of Reduced Transport Fluid (RTF medium) in sterile vials and transported to the microbiology laboratory. Samples were subjected to DNA extraction using Tris-EDTA buffer (TE buffer). Polymerase Chain Reaction (PCR) was performed using species-specific primers targeting the 16S rRNA gene of: *Porphyromonas gingivalis*, Prevotella intermedia, *Aggregatibacter actinomycetemcomitans* Amplified products were run on 2% agarose gel electrophoresis with ethidium bromide staining. A DNA ladder was used as a molecular weight marker. Presence of specific bands confirmed the target organisms. Following full-mouth scaling and root planing, participants were recalled after 1 week for irrigation. Subgingival irrigation procedure: A disposable 5 mL syringe with a blunt-end needle was used. 5 mL of solution (either CHX or Propolis) was slowly deposited for 30 seconds into each selected periodontal pocket at six sites (mesiobuccal, buccal, distobuccal, mesiolingual, lingual, distolingual). Oral hygiene instructions were reinforced and participants were instructed to brush twice daily with a soft-bristle toothbrush. No adjunctive antimicrobial mouth rinses were prescribed during the study period. Baseline (Day 0): Clinical parameters + microbiological sampling. Day 15: Clinical parameter recording. Day 30: Clinical parameter recording + microbiological sampling. "Data was entered into Microsoft Excel and analyzed using SPSS version XX (IBM Corp.). Descriptive statistics were expressed as mean ± standard deviation (SD). Intra-group comparisons: Paired t-test (baseline vs follow-up)". Inter-group comparisons: Independent t-test / ANOVA. Microbiological data: Chi-square test to compare presence/absence of pathogens. Level of significance: p < 0.05 considered statistically significant.

## Results:

The mean plaque index values in both groups showed a gradual reduction from baseline to 30 days. In the control group (Group A), PI decreased from 2.25 ± 0.42 at baseline to 1.56 ± 0.28 at 30 days, while in the test group (Group B), it reduced from 2.28 ± 0.37 to 1.60 ± 0.30. Although the mean reduction was slightly higher in the control group, there was statistically non-significant difference at any time point (P > 0.05). This indicates that both interventions effectively reduced plaque accumulation over time, but with no significant difference between them (Table 1 - see PDF, Figure 1 - see PDF). The gingival index values demonstrated a marked reduction in both groups over the study period. In Group A, the GI declined from 2.79 ± 0.18 at baseline to 1.15 ± 0.26 at 30 days, whereas in Group B, it decreased from 2.73 ± 0.21 to 1.20 ± 0.31. The percentage reduction was greater in Group A, but the intergroup comparison showed no statistically significant differences (P > 0.05) across all intervals. These findings suggest that both groups experienced significant improvement in gingival health, with comparable efficacy between the two treatment modalities (Table 2 - see PDF, Figure 2 - see PDF). The probing pocket depth showed considerable improvement in both groups over the 30-day period. In Group A, the PPD reduced from 6.48 ± 1.02 at baseline to 3.62 ± 1.14 at 30 days, while in Group B, it decreased from 6.15 ± 0.98 to 4.05 ± 1.09. The mean difference between groups was not statistically significant at baseline or at follow-up intervals (P > 0.05). However, the overall trend indicates that both groups benefited from treatment, with slightly greater pocket depth reduction observed in the control group (Table 3 - see PDF, Figure 3 - see PDF). The percentage reduction in PI, GI and PPD was statistically significant within both groups across all time intervals. For PI, the maximum improvement was observed between baseline and 30 days (30.95% in Group A; 29.10% in Group B). Gingival index values showed the most pronounced improvement, with reductions of 59.20% in Group A and 54.60% in Group B from baseline to 30 days. Similarly, PPD reduced by 42.95% in Group A and 34.10% in Group B over the same period. All intragroup changes were statistically significant (P < 0.05), indicating substantial clinical improvement, though intergroup differences remained insignificant (Table 4 - see PDF, Figure 4 - see PDF). The microbial analysis demonstrated a significant reduction in colony-forming units from baseline to 30 days in both groups. Group A showed a decrease from 1.48x10^3^ ± 140.62 to 7.2x10^1^ ± 15.10, while Group B reduced from 1.460x10^3^ ± 133.45 to 8.0x10^1^ ± 14.25. Intragroup comparisons were highly significant (P < 0.0001), confirming effective microbial control. However, intergroup differences were not statistically significant (P = 0.1850 at baseline and P = 0.1052 at 30 days), indicating that both groups achieved similar levels of bacterial reduction (Table 5 - see PDF, Figure 5 - see PDF).

## Discussion:

In this randomized single-blind study, both the 0.2% chlorhexidine (CHX) and 25% propolis extract groups showed significant improvements in clinical parameters (Gingival Index (GI), Plaque Index (PI) and Probing Pocket Depth (PPD)) after 15 and 30 days compared with baseline. However, the propolis group demonstrated greater reductions in GI, PI, PPD and bacterial detection rates of *P. gingivalis*, *P. intermedia* and *A. actinomycetemcomitans* at day 30. These results suggest that 25% propolis subgingival irrigation may be at least as effective, if not slightly superior, to 0.2% chlorhexidine as an adjunct to scaling and root planing (SRP) in patients with periodontal pockets between 4-6 mm. The antibacterial properties of CHX are well-known, as it works by disrupting bacterial cell walls and altering cell permeability, which leads to the precipitation of cytoplasmic components. While it is effective in controlling plaque and reducing gingival inflammation, its limitations include poor penetration into deep periodontal niches, making it less effective in completely eliminating subgingival pathogens after SRP. This may explain why in our study, although CHX was effective, residual bacteria were still present in tissue niches, leading to slower or less complete recovery. These findings are consistent with earlier studies, such as those by López-Valverde *et al.* (2021) [13], who highlighted the role of CHX in short-term adjunctive periodontal therapy, particularly in reducing bleeding on probing and plaque accumulation. In contrast, propolis offers broader bioactivity through its antimicrobial, anti-inflammatory, antioxidant and immunomodulatory properties. Propolis disrupts bacterial cell walls and inhibits bacterial enzymes while also modulating host inflammatory responses. Studies, including Seth *et al.* (2022) [14], have shown that propolis is as effective as CHX in improving clinical and microbiological parameters when used as a subgingival irrigant, with the added benefit of modulating the host's inflammatory response. This dual action-direct antimicrobial activity and host modulation-may explain the more pronounced clinical improvements observed in our study.

Zarch *et al.* (2021) [15] found that propolis significantly reduced matrix metalloproteinases (MMPs), which are implicated in periodontal tissue degradation. This host-modulatory effect of propolis suggests that it may not only control microbial growth but also mitigate the tissue-damaging effects of inflammation. This is further supported by Sahu *et al.* (2023) [16], who demonstrated that subgingival propolis nanoparticles provided sustained release and enhanced bioavailability, leading to improved clinical outcomes, including reductions in PPD and bleeding on probing (BOP). Our study concurs with these findings, showing that propolis extract may offer additional tissue-level benefits, potentially explaining the superior clinical outcomes observed. Moreover, studies such as Waqar *et al.* (2024) [17] have compare propolis mouthwashes with CHX and reported stronger reductions in BOP and sustained clinical improvements with propolis. In a recent study, Pezzella *et al.* (2025) [18] highlighted the importance of functional biomaterials in various medical applications, emphasizing their potential in improving patient outcomes and advancing biomedical research. Moreover, Alghutaimel *et al.* (2024) [19] conducted a comprehensive review on dental materials, examining their impact on clinical practices and the effectiveness of current innovations in the field. This aligns with our observation that propolis produced greater reductions in GI and PI, suggesting its potential for long-term clinical benefits. As demonstrated by Patel *et al.* (2022) [20], a 25% propolis extract used for sub-gingival irrigation following scaling and root-planing showed similar improvements in periodontal indices to 0.2% chlorhexidine, indicating its potential as an adjunctive treatment for chronic periodontitis. Additionally, the safety profile of propolis, with minimal side effects, makes it a desirable natural alternative, especially for patients who experience adverse reactions to CHX. Taken together, these studies underscore the dual benefits of propolis-both its antimicrobial and anti-inflammatory effects-making it a promising natural adjunct to SRP. Propolis may offer a more holistic approach to periodontal therapy, addressing both the microbial and inflammatory components of the disease. While CHX remains a staple in periodontal care, propolis presents a viable alternative, especially in patients seeking natural treatments or those with CHX intolerance. Larger trials are needed to confirm these findings and further explore the long-term benefits of propolis in periodontal therapy.

## Limitations:

Small sample size (n = 20) - the trial was powered for medium effect sizes, but slight differences might not be detected and external validity is limited. Short follow-up (30 days) - The periodontal healing and recolonization dynamics extend beyond one month; a longer follow-up (3-6 months) would better capture the sustained effects on PPD and attachment. Single-center and single geographic source of propolis - propolis chemical composition varies with botanical source; results may not be generalized to other propolis preparations. Single application protocol - only a single irrigation episode after SRP was performed; repeated applications or other delivery systems (gels, nanoparticles) might yield different outcomes. PCR qualitative reporting - PCR was used for detection of target pathogens (presence/absence). Quantitative PCR (qPCR) or CFU counts would provide better assessment of bacterial load changes. Lack of blinding for operators - while participants were blinded, the operator performing irrigation could not be blinded to the solution used; this introduces potential performance bias (although separate examiners for recording minimized measurement bias).

## Conclusion:

In this randomized single-blind trial, subgingival irrigation with 25% propolis extract showed promising results as an adjunctive subgingival irrigant, with superior short-term outcomes compared to chlorhexidine. Larger and longer-term studies are required to confirm these findings.
